# Monthly Summary

**Published:** 1891-11

**Authors:** 


### Monthly Summary.

IS DENTISTRY A DANGEROUS PROFESSION? A REPLY. —BY —— L. D. S.—Under the above heading Dr. Dudley W. Buxton discusses very ably and very kindly, some of the incidents in the daily routine life of the dentist which he thinks have an adverse re-action upon his health and comfort.

He takes as his example, the man who full of work, is allowing it to absorb his whole being, and gradually kill him, or at all events make his life burdensome by reason of his success. But there is perhaps, here and there, a case of insomnia, of neurasthenia, or nervous irritability, which arises from anxiety and care—the anxiety of not knowing where your next patient is to come from, how you are to pay your rent and taxes; how you are to provide for your wife and increasing family—a family perhaps not increasing in number but in expensiveness, and especially so since the demand for a higher dental education causes one to scan the tables of Hospital and lecture fees with grave consideration. The Dental Act will, we hope, in time work miracles for the next generation, but it does not do much to protect us from the advertising and cheap practitioners who infest our towns. We who have our L.D.S. diploma, and belong to the British Dental Association, must at any rate keep ourselves respectable, live in good houses, abhor all that which should be despised as unprofessional, while we sit and moan that our practices are being filched away, and our incomes growing less. I have been frequently told so by some of the older practitioners at our B. D. A. meetings, and I believe it to be perfectly true.

A few years ago, a traveller for a highly respectable dental supply house told me he had been requested by the firm to call upon the chemists in the town and suburbs, to solicit their orders, but he thought it was a waste of time to do so. On returning to London, he was asked how it was he had not obeyed his instructions, and he replied he could not afford the time, and it was of little use. He was at once informed that the chemist was the dentist of the future, and unless he did look them up they (the firm) would be compelled to get another traveller.

I ceased to do business with that firm on the death of that loyal traveller, but have little doubt the houses all make it a purely business concern, and supply the just and the unjust with their richest blessings with equal politeness.

It is hard—very hard lines for a man, who is a capable dentist, perhaps holding many public appointments to be obliged to see himself thus being elbowed out of the way, in order that he may maintain the status of his profession. No ride on a cycle, or horseback or walk before breakfast will heal his nervous irritability, and he simply dreads to ask for his bank-book. Six or eight hours hard work in his surgery, or work room, would be the finest tonic for him provided he got his fees down when the work was completed—and did not have to wait for years, and finally fight for them in the Connty Court. When I think of the wonderful progress Dentistry has made, or is said to have made as a profession at our after dinner meetings, when we are being flattered by our guests, and we feel like millionaires basking in the sunshine, I sometimes wonder if we have not been going it too fast, and if, after all, in the sweet bye and-bye the public will not stick to the man who can do his work, rather than run after the newly-fledged chicken of many letters, and more science than practical knowledge. I suspect many dentists, who in the nature of things, would have liked their sons to have succeeded them, and who have ample opportunities for teaching them all the really essential parts of their profession, will not be able to afford the purely ornamental adjuncts, of which we are hearing so much just now—the peacock's feathers—and will be driven to find other and less expensive occupations for them—or on the other hand if they determine to go the whole hog, come what come may, will eat the bread of carefulness, and sleep the night with open eyelids. This all seems a mournful dirge of dental neuralgia—but I doubt not, and I am sorry, that it will find many an echo—it is the same with the medical—the same with the clerical professions—we are hanging ourselves with restrictions, and we cannot afford to pay for the rope. By all means let us have the Provident and Sick Fund—Dudley Buxton suggests, let us try and keep off the Benevolent Fund, and help it, and let us keep our spirits up, but after all it is the pneumogastric nerve which has a lot to do with the "daugerous profession of a dentist."—*Dental Record.*

---

DENTISTRY IN FRANCE AND IN AMERICA.—Dr. J. H. Spaulding, a former Minneapolis dentist, who has been practicing in Paris for the past five or six years, says: In France, the patient places himself more under the care of the dentist; he goes to him to save his teeth, and is anxious to have him take whatever course he thinks best. Americans, on the other hand, are apt to be dictatorial, and are often given to grumbling and finding fault with the dentist's decision. The Frenchman has a greater respect for authority and professional skill.

On one point, however, the men and women of France are firm. They will not have their front teeth filled with gold. They object to wearing conspicuous jewelry in their teeth, and insist on having none but white filling where it will show. The gold filling in the teeth of Americans is a source of no little amusement to them. They suppose it is a fad among Americans to have the brightest gold possible in their front teeth; and some of them actually believe that American women have their teeth set with diamonds.

The Frenchman's preference for amalgam and cement fillings is entirely irrespective of the cost of the materials. The French dentist charges for his services without regard to the material used. The minimum fee is the same for gold, cement and amalgam fillings, and in long operations the charge is regulated entirely by the time required.

A feature of the practice in France, which is very agreeable to the dentist, is the manner in which the patient pays his fee. The Frenchman, when he settles with his dentist, thanks him for his kindness, attention and delicate manner of operating. An American is apt to think he has done all that can possibly be expected of him when he has drawn a check for the amount of the bill. He regards the affair as a commercial transaction, which consists largely in buying so much amalgam or gold, and overlooks the intimate personal relation between himself and his dentist. In France, the manifestation of kindly feeling between dentist and patient extends to the exchange of invitations and other social civilities; and this, too, in a country where it is difficult to gain entrance to good society. The Frenchman is loyal to his dentist professionally, and in case of removal will patronize his successor, or some one whom he recommends.

Though there are a few lady dentists in France, and more in Russia and Germany, the young lady assistant so common with American dentists is rarely found. Dr. Spaulding has one in his office, and some of his patients have expressed themselves pleased with the innovation, but others object to having a third person around. The patients sometimes insist that no one shall see them at the office. Europeans in general, and especially among the less favcred classes, neglect their teeth much more than Americans do.

The French dentists have a hig opinion of the skill and inventive ability of their American brethren, though they consider them a little pretentious and showy in some of their methods. In the manufacture of plates, the French dentist excels the American, as that branch of the profession is developed into a specialty, and not leit to apprentices and students, as in America.

"There is no other profession on the face of the earth," says Dr. Spaulding, "in which the productive years are so short as in dentistry, or the demand on the vital forces so severe and exhausting. The physician is in demand when he is fifty, but if the dentist's hand shakes a little, or if the people imagine that he can't see as well as he once did, he is done for. When he is ripe in experience, he is about ready to break down. People who grumble at their dentist's fees, should remember that they are paying for ten or fifteen years of a man's life"—*Items of Interest.*

---

MAKING AN ARTIFICIAL SET OF TEETH.—We ought seldom to extract teeth, but for many reasons they *are* lost and substitutes must be provided. Though ordinarily these are not difficult to manage, occasionally they tax the ingenuity to the utmost. To obtain a really good impression is often difficult. Indeed, impressions for entire dentures are generally imperfect. It is usually claimed that partial impressions are the most difficult, but I believe this to be a mistake.

The best material for all cases is no doubt plaster. I cannot conceive of any condition, where an impression can be secured at all, that plaster will not be best. Some months since, a gentleman came to me for a partial plate to supply the four upper incisors and one first bicuspid. The arch was high and narrow, teeth long, loose and bell crowned; the lower teeth very long and irregular, and jaws incapable of opening wide enough for the impression cup. It seemed a "desperate" case.

First, strips of wax were placed against the lingual surfaces of the teeth, which had been previously dried. A piece of flexible card-board was cut to fit within the arch; plaster mixed quite thick, but so as to harden slowly, was then carried up into the highest part of the mouth and pressed up with the finger, then more applied to all the parts desired to have reproduced, and lastly the card-board pressed up and held in place while the plaster hardened; removal was not difficult, and a good impression resulted.

For obtaining a full impression, an examination of the mouth should always be the first move, and the finger introduced, not only to ascertain the condition of the tissues, but to try the ability of the soft palate to endure the presence of a foreign substance. When symptoms of revolt are manifested, the finger should be held in contact with the palate for some moments, the patient in the meantime being encouraged to exercise self-control; then a suitable tray selected, a piece of wax molded across the posterior palatal portion, and this introduced and held in position for a short time to further accustom the patient to the operation. A few earnest words should be spoken to impress on the patient the necessity for co-operation and the importance of securing a good impression.

These preliminaries need occupy but a few minutes, and will often obviate the necessity of repeating the operation before securing the desired result, or the worse alternative, of accepting an imperfect and uncertain impression. The plaster now being carefully mixed, the patient sitting in an upright position, the tray is introduced, the posterior portion pressed up first and then the tray forced upward and slightly backward with slow, steady movement. Excessive protrusion of the alveolar process, especially when accompanied by a short lip, is a problem for the solution of which several plans have been offered; first, removal of the process; second, use of plain teeth placed directly against or beneath the ridge; third, plain teeth mounted with a thin rim of pink rubber; fourth, gum sections selected, ground and adjusted as closely as possible to the natural gums. The first or heroic plan is undoubtedly the best, but is seldom practiced because of the timidity of the patients; the second is objectionable because of danger of shrinkage of the alveolus, and because such work lacks strength. The third is inadmissible because of the lack of support afforded by pink rubber, and on account of the want of natural appearance, for pink rubber resembles nothing on the earth, except, perhaps, another piece of the same material. The use for which it seems best adapted is for packing joints in making gum sets, and it is certainly superior to any perishable material for that purpose.

The fourth plan seems to me the best, and appropriate gum sections will generally give good results, though not always what we might desire. Perhaps the time may come when dentists will have advanced so far in skill and ambition that each shall possess a furnace, and construct porcelain work to meet all requirements.—*Dr. G. W. Dennis in Illinois Society.*

---

SAVING A NERVE.—The following instance of saving a nerve is given by Dr. Morrison, of St. Louis:

It was my misfortune, in 1861, to have a bicuspid exposed on its distal surface in its excavation. The dentist in charge of the case decided that I had better have the tooth extracted, as the pulp was badly exposed; that it was impossible to put a filling of any kind upon it; that if the pulp was removed the tooth would be destroyed and lost in a very short time, and that I had better submit to its extraction immediately. I called a halt on that.

A year or two before that, Dr. Atkinson, of New York, recommended the capping of exposed pulps with oxychloride of zinc. The excavation of the tooth was a painful proceeding, but after a consultation it was decided to cap it with oxychloride. I sat under the operation, and did not have much to say, but wanted to have the tooth preserved if it could be done. They made a paste of the oxychloride after making a dressing of old-fashioned German wood creosote and applying it to the cavity. The oxychloride capping was applied, and it was one of the most intensely painful operations I ever experienced. I nearly turned a summersault out of the chair, and came very near going out through the window. A little while after that a gold filling was made over the oxychloride capping, and that filling I wore for eight or nine years, and that tooth has always been sensitive to thermal changes. Eight or nine years after I had it refilled with gold, the dentist who performed the operation, instead of making a good contour filling, made a flat filling, and there was obstruction to cleansing or cleaning the tooth with a toothpick at cervical space. I had that gold filling removed, and another one put in its place. This work was done so thoroughly that a fracture occurred in packing the filling in, and the tooth was sensitive for more than two years. After that every time I closed my teeth I felt there was something wrong with it. Nearly every dentist whom I could interest in the matter examined it, and I got the opinion of each one. It was pronounced all right; at the same time I knew there was something wrong. One evening while eating some oranges the inside cusp of the tooth came away, leaving it much more comfortable. I had the remaining external cusp ground shorter, and I am now wearing a metal shell over the tooth. It was capped thirty years ago; it was then condemned and said to be beyond all hope of preservation; it is alive to day; it is not so sensitive to thermal changes, because there is such a large metal coat over it.—*Dental Review.*

---

WAS COCAINE THE CAUSE OF THIS DEATH?—Miss X. called on the 7th of August to make an appointment for the following day, Friday, at seven o'clock, A. M. On that day I proceeded to extract two superior teeth, after having made two external and one internal subcutaneous injections of a solution (cocaine) of 1 to 100—*au centieme*; the teeth were very loose and the success was complete. After several minutes my patient expressed a wish to have another tooth in the left inferior jaw, and equally as loose as the others, extracted. I injected the rest of the cocaine in my syringe, and after waiting a few minutes extracted the tooth. The wound did not bleed. I gave the patient a little warm water and a little black coffee to induce the flow of blood. Nothing appeared to indicate an accident; the patient seemed to be all right, although somewhat fatigued. I left her in the care of her uncle while I went for more coffee.

On my return the young lady had fainted. She was placed on the floor, her clothlng loosened, dashes of cold water, friction,—in short, everything was done that the situation indicated. The physician, hastily summoned, continued the same treatment, and, in addition, gave nitrate of amyl, oxygen, and subcutaneous injections of ether; but the patient succumbed after a half hour of unsuccessful treatment.

The physician who made the death certificate, after describing the fainting of the patient, concluded that death was caused by cocaine, as that salt had been employed.

Was cocaine the cause of death? I will prove the contrary.

When the honorable doctor incriminated cocaine he was ignorant, as I was myself, at the time, that a large knotted cord, called *corde a lessive*, was wound four times around the body of the girl, compressing the thorax. This cord was drawn so tightly that its presence was not suspected and could not be cut without injury to the skin.

This explains why the attempts at artificial respiration were unsuccessful.—M. BOUCHARD, in *International.*

---

HAPPY THOUGHT.—One day, in 1830, when a working jeweler, Joseph Gillott, now the famous steel pen maker, accidentally split one of his fine steel tools, and being suddenly required to sign a receipt, not finding his quill pen at hand, he used the split tool as a ready substitute. The happy accident led to the idea of making pens of steel.

